# Dr. Abraham F. Crans

**Published:** 1909-11

**Authors:** 


Dr. Abraham F. Crans, of Olyphant, Pa., died at his home in that village, of heart disease, October 2, 1909. Dr. Crans, formerly lived in Owego, N. Y., where he had been health officer and trustee of the town. He graduated at the Medical Department of the University of Buffalo in 1890.

